# Study on a new type of environment-friendly polymer and its preliminary application as soil consolidation agent during tree transplanting

**DOI:** 10.1038/s41598-021-83594-2

**Published:** 2021-03-10

**Authors:** Shaoli Wang, Donglu Wei, Xuping Yang, Shengju Song, Lifang Sun, Xuebing Xin, Guangshun Zheng, Ran Wang, LiLi Liu, Jingshuang Sun, Haixia Wang, Fuling Lv, Wenjuan Mo, Hong Wang, Chaoxing Luo, Zhengqi Xiong, Shaobo Wang, Shaofeng Li, Yongxiu Xia

**Affiliations:** 1 Experimental Center of Forestry in North China, Chinese Academy of Forestry, Beijing, 102300 China; 2 College of Material and Chemical Engineering, Heilongjiang Institute of Technology, Harbin, 150050 China; 3 R & D Center, China Academy of Launch Vehicle Technology, Beijing, 100076 China; 4 Foreign Language Teaching and Research Press, Beijing, 100089 China

**Keywords:** Chemical engineering, Polymers

## Abstract

Transplanting trees with rhizospheric soil is an important way to facilitate tree survival in the process of landscaping and reforestation. Traditional way to prevent looseness of rhizospheric soil is forming soil balls around the roots with bags, boxes or rope wrapping, which is cumbersome, laborious and easy to break. This study is aimed to develop a new type of degradable environment-friendly polymer as soil consolidation agent to facilitate tree transplanting. In this paper, the KGM/CA/PVA ternary blending soil consolidation agent was prepared by using Konjac glucomannan (KGM), chitosan (CA) and polyvinyl alcohol (PVA) as raw materials. Through the verification and evaluation, the clay and sandy soil can be consolidated and formed into soil balls by the ternary blend adhesive, which was convenient for transportation. The preliminary application of the ternary blend adhesive in the transplanting process of sierra salvia, Japanese Spindle (*Euonymus japonicus*) and *Juniperus sabina* ‘Tamaricifolia’ confirmed that the application of soil consolidation agent can effectively solve the problem that the root ball of seedling is easily broken in the process of transplant. And the application of soil consolidation agent has no adverse effect on the growth of transplanted seedlings. The research and development of ternary blending soil consolidation agent and its preliminary application in seedling transplanting will provide a new solution to solve the problem of soil ball breakage in the process of seedling transplanting. This is an important stage in the development of new seedling transplanting technology. Therefore, the research and development of soil consolidation agent is of great significance.

## Introduction

With the aggravation of air pollution in modern cities, the increase of urban trees will improve the urban ecological environment and improve the quality of life^1^. In the urban greening work, the survival rate of transplanted seedlings is one of the important criteria for evaluating the quality of afforestation and land greening projects. There are many factors affecting the survival of transplanted seedlings, such as seedling pruning^2^, planting depth^3^. But, at present, the method of seedling transplantation with mother soil has the highest survival rate in nursery transplanting. However, the seedlings, especially large seedlings (or big trees) lifted with soil ball, is a challenging task. The nursery lands are mostly loose soil having poor agglomeration ability, so that digging up the earth ball is a high skill, and making the ball wrapped, tied or packed in boxes for transportation is time and energy consuming. Even so, the breakage of soil balls frequently occurs in the process of seedling loading and transportation. In terms of urban and rural greening, soil ball breaking is the main reason for the death of large seedlings (or large trees) after their transplantation.

Therefore, in order to ensure the integrity of soil balls, first of all, we should change the loose characteristics of soil at seedling roots. Can we develop a substance that can consolidate the loose soil at the seedling roots for handling and transplantation, and then let the solid soil bulk collapse and dispersed into their new sites by itself after the seedlings planting? This will not only solve the problem of the difficulty of lifting seedling with loose soil balls, but also avoid the breakage of soil balls during the loading and unloading steps. At the same time, it will save a lot of packaging materials in spite of plastic bags, straw ropes or wooden boxes. Nowadays, there is nowhere to live without polymer materials, and the application of polymers could be extended to the ecological environment engineering of forestation and greening. It is quite reasonable to use some kinds of polymers in the seedling transplantation in the aspects that we just specified.

For the consolidation of soil balls with the help of polymers for further transportation and transplantation of seedling, three points should be considered: first, the polymer-type soil consolidation agent does not impair the growth of seedlings; second, the polymer consolidation agent solidifies fast enough after spreading on the soil ball for seedling handling and transportation; and third, these steps are easy, simple and convenient.

At present, soil stabilizers rapidly solidifying soil are those mainly used in the treatment of building foundation, highway sub-grade reinforcement, water conservancy engineering such as slope solidification, canal seepage prevention and so on^4–11^. Their task mainly aims at solidifying soil as long as possible, better for permanent, i.e. unidirectional solidification without further collapse^4–14^. Furthermore, the inorganic soil solidifying agents have a seriously negative effect on the soil. The solidified soil layer basically loses the ability for plant growth, and its alkaline substance rinsed into the land is harmful to the vegetation. At the same time, the treatment of abandoned solidified soil is also difficult^7–14^. It is clear that the soil stabilizer used in construction can’t be used for the consolidation of seedling soil. Therefore, in order to afforestation, it is necessary to study the consolidation agent that is suitable for both the seedling soil consolidation and seedling growth after transplantation.

The essence of soil consolidating agent is the adhesive that can bond the soil. There are many kinds of adhesives. The main types of adhesives are solvent-based type, hot–melt type and water-based type^15–28^. Solvent based adhesives use volatile organic compounds as solvents, such as benzene and toluene. These solvents will directly volatilize to pollute the environment and harm the health of human body^15–19^. For the environmental protection our soil consolidation agent should not pollute the soil and groundwater and neither impair normal growth of the seedlings. Therefore, solvent-based adhesives can’t be the candidate. The existing hot–melt adhesives have poor wettability and are difficult to be spread evenly because of their high viscosity after melting, so that they are not suitable for large area coating and bonding^20–23^. It is clear that the existing hot–melt adhesives are not candidates for the consolidation of seedling soil. Water adhesives are a group of environment-friendly adhesives, which takes natural and synthetic polymers as sticky materials, and uses water as solvent or dispersant instead of toxic organic solvents. The main advantages of water-based adhesives are no poison, no pollution, no burning, safe use and easy to realize clean production technology. Their main disadvantage is the slow drying rate of the film at low temperature and high humidity. And their water resistance and frost resistance are poor^24–28^. Therefore, it is necessary to strengthen their water resistance and accelerate their drying speed if they will be used to consolidate soil for seeding transplantation.

The ideal soil consolidation agent should not only have strong adhesion in wet environment and reach certain strength for handling, but also degrade fast enough within a given period of time. Their degradation products should be non-toxic and harmless, i.e. do not pollute soil and groundwater, and do not cause disease to plants. Therefore, the soil consolidation agents to be prepared should be a kind of biodegradable adhesives. Although there are many kinds of biodegradable adhesives, they can’t be directly applied to the consolidation of seedling soil. In order to avoid the crushing of soil balls in the whole process of seedling transplanting, to ensure the supply of nutrient to the seedling roots, and to increase the survival rate of the transplanted seedlings, it is necessary to study the composition and properties of biodegradable polymers, to design and synthesize the chemical structure of the candidate polymers, to characterize their structure and properties, to explore the degradation mechanism of these polymer consolidation agents and their influence on the growth of seedlings.

Konjac glucomannan (KGM) is the main component of Amorphophallus in Araceae. KGM is a non-ionic water-soluble polymer polysaccharide. It is a compound polysaccharide formed by combining D-glucose and D-mannose through β-1,4-pyranoside bonds. Because of its excellent properties, such as good cross-linking, film-forming and gelling properties, good biodegradability, hygroscopicity and water permeability, and low toxicity^29–31^, konjac glucomannan has become an ideal raw material for the preparation of degradable soil consolidation agents.

Chitosan (CA) is a natural cationic polysaccharide obtained by deacetylation of chitin. It is the only natural alkaline polysaccharide in nature and the most important derivative of chitin. Chitosan has many unique physical and chemical properties and biological activities, such as cationic polyelectrolyte, multi-functional group reaction activity, antibacterial, biocompatibility and degradability. Its molecular structure contains amino, hydroxyl, acetylamino and other active groups, which can produce various chitosan derivatives with different physical, chemical properties and biological functions. It can be found from previous reports that although the blending of KGM and CA can effectively improve the mechanical properties of the KGM polymer, its water resistance needs to be improved.

Polyvinyl alcohol (PVA) is a kind of white powder water-soluble polymer resin obtained by hydrolysis of polyvinyl acetate. It is non-toxic, harmless, low price, good film forming ability and high strength of the adhesive film. PVA can improve the adhesion and moisture resistance when used as an auxiliary of adhesive. Therefore, PVA is the best raw material to improve the water resistance of KGM / CA blend polymer.

Therefore, in the present study biodegradable materials of KGM, CA and PVA were used as the main raw materials. Through polymer blending technology, the polymer-type consolidation agents were prepared and used to make samples of consolidated soil. The feasibility of the application of ternary blend adhesive on transplanting seedlings was confirmed in the aspects of the viscosity of the ternary blend adhesive, the structure of KGM/CA/PVA ternary blend membrane and the surface morphology, compression resistance and vibration resistance of the consolidated soil ball. Most importantly, the preliminary application of the ternary blend adhesive in the transplanting process of sierra salvia, Euonymus japonicus and Juniperus sabina ‘Tamaricifolia’, confirmed that the application of soil consolidation agent can effectively solve the problem that the root ball of seedling is easily broken in the process of transplant.

Although the research on the ternary blend soil consolidation agent in this paper is the primary stage to solve the problem of soil ball breakage in the process of seedling transplanting, it is an indispensable part of the development of new seedling transplanting technology. Therefore, it is of great significance to study the preparation of ternary blend soil consolidation agent and its application in seedling transplanting.

## Results

### Viscosity of polymer adhesives and influencing blending factors

Polymer adhesives used as the soil consolidation agent should have their viscosity high enough to adhere well on the soil ball and later to keep the root ball strong. Their preparation conditions were investigated as follows.

**Figure 1:**
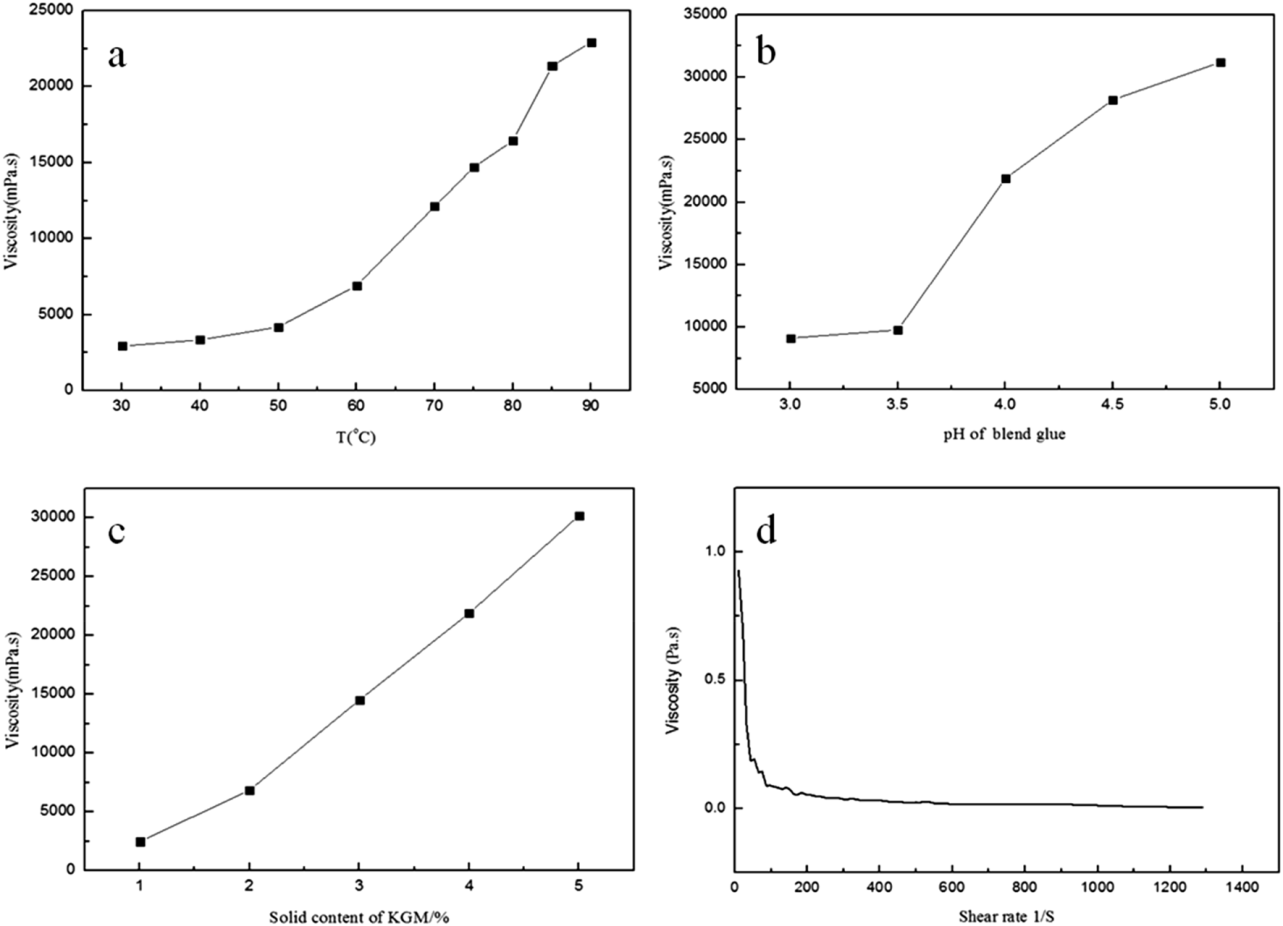
Relationship between blending factors and viscosity of ternary blend adhesive. (**a**) General conditions: Stirring time-40 min; Content of KGM, CA, PVA = 4.0%, 4.0%, 5.0%; pH of adhesive-4.5; (**b**) General conditions: Stirring time-40 min; T-85℃; Content of KGM, CA, PVA = 4.0%, 4.0%, 5.0%; (**c**) General conditions: Stirring time-40 min; T-85℃; Content of PVA-5.0%;pH of glue-4.5; (**d**) General conditions: Stirring time-40 min; T-85℃; Content of KGM, CA, PVA-4.0%, 4.0%, 5.0%; pH of glue-4.5. The test T of Rheological diagram-25℃.

The blending temperature had a certain effect on the viscosity of the ternary blend adhesive, as shown in Fig. 1a. The viscosity of the ternary blend adhesive gradually increased with the increase of blending temperature, and the higher the temperature, the viscosity increased more significantly. Especially, when the blending temperature was 85℃, the viscosity grew fastest. After that the growth trend slowed down. Through studying the influence of pH on the viscosity of the ternary blend adhesive, it was found that pH had a great influence on the viscosity of the adhesive, as shown in Fig. 1b. With the increase of pH, the viscosity increased. When the pH of the adhesive reached 6, a yellow gel appeared before cooling, and was viscous with a poor flowability. After cooling to room temperature, the adhesive became a “jelly gel”. This phenomenon indicated that with the increase of the pH, a large number of intramolecular and intermolecular hydrogen bonds were formed among –NH_2_, –OH on the molecular chains of CA and –OH on the molecular chains of KGM. From this result the pH of the glue should be used under acidic condition, say at pH lower than 5. The solid content of KGM in the glue had a significant effect on the viscosity of the ternary blend adhesive, as shown in Fig. 1c. With the increase of solid content of KGM, CA and PVA, the viscosity of ternary blend adhesive increased linearly. For the practical application, a balance between the solid content and the viscosity should be chosen. From the steady-state rate scanning diagram at a constant temperature, it is clear that the blend glue was a pseudo-plastic fluid (Fig. 1d). With the increase of shear rate, the apparent viscosity of the adhesive decreased sharply at the start and then gradually. This shear-thinning behavior was beneficial to the spreading of the ternary blend adhesive onto the soil column.

### The structure of KGM/CA/PVA ternary blend membrane

**Figure 2:**
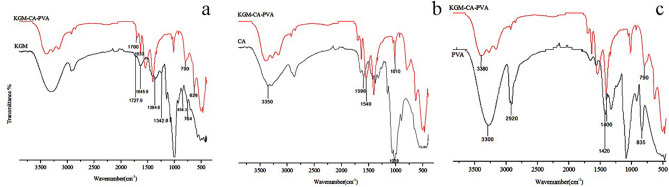
IR spectra of KGM/CA/PVA, KGM, CA and PVA.

The interaction of KGM, CA and PVA in the ternary blend adhesive was analyzed by observing the difference between infrared spectra of KGM/CA/PVA ternary blend membrane and those of three components, as shown in Fig. 2. Comparing IR spectra of raw material KGM, CA and PVA with the IR spectra of KGM/CA/PVA, it can be seen that the absorption peaks of KGM in the ternary blend adhesive at 1727.9, 1645.9, 858.3 and 764 cm^−1^ all shifted slightly (Fig. 2a). Second, the absorption peak of CA in the crystallization sensitive zone 1028 cm^−1^ has changed significantly in the blend membrane. The absorption peak at 1028 cm^−1^ became an inseparable peak in the blend membrane, which further indicated that there was a strong interaction between CA and KGM molecules in the blend membrane and interfered with the original crystal structure of CA. The absorption peak of CA in ternary blend adhesive shifted from 1598 to 1540 cm^−1^ (Fig. 2b). In addition, the PVA deformation peak of –CH_2_ at 1420 cm^−1^ changed obviously with blending. Compared with the three raw materials of KGM, CA and PVA, the characteristic peaks of the ternary blend adhesive at 3380 cm^−1^ changed from larger wide peaks to smaller spikes, which indicated that –OH and –NH were reduced, and hydrophilicity was weakened (Fig. 2c). These facts fully illustrated the strong interaction of hydrogen bonds in ternary blend adhesive.

### Moisture permeability of polymer blend membranes

When the KGM/CA/PVA ternary blend adhesive is used to consolidate soil to form soil balls, certain moisture permeability is required to ensure water reaching seedling roots during the whole process of the transplantation. Therefore, it is important to investigate the moisture permeability of KGM/CA/PVA ternary blend membranes. As on the viscosity of KGM/CA/PVA blends, blending temperature, pH and solid content of the glue for the preparation also directly affected the moisture permeability of the blend film.

**Figure 3:**
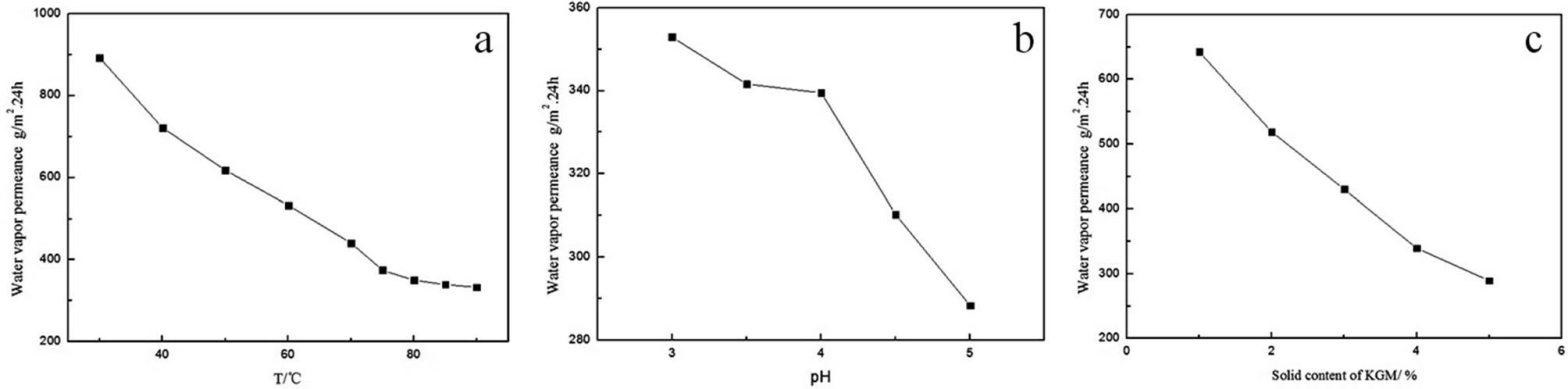
Influence of blending conditions on water vapor transmission. (**a**) General conditions: stirring time-40 min; Content of KGM, CA, PVA = 4.0%, 4.0%, 5.0%; pH of glue-4.5; (**b**) general conditions: Stirring time-40 min; T-85℃; Content of KGM, CA, PVA = 4.0%, 4.0%, 5.0% ; (**c**). General conditions: Stirring time-40 min; T-85℃;KGM:CA = 1:1; Content of PVA-5.0%; pH of glue-4.5.

The blending temperature had a great influence on the vapor permeability of the blend film. With the increase of blending temperature, the vapor permeability of KGM/CA/PVA ternary structure was obviously reduced. When the blending temperature was 85℃, the vapor permeation volume tended to be stable, as shown in Fig. 3a. The pH of the glue had a certain effect on the vapor permeability of the blend membrane. With the increase of pH, the water vapor permeability of the blend membrane gradually decreased, as shown in Fig. 3b. The solid content of the adhesive had a certain effect on the water vapor permeability of the blend membrane. With the increase of the solid content of the adhesive, the water vapor permeability of the adhesive membrane decreased linearly, as shown in Fig. 3c.

### Surface morphology of consolidated soil balls

**Figure 4:**
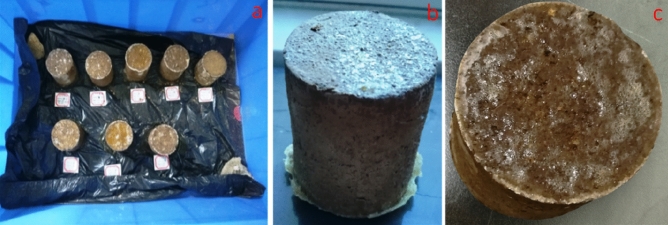
The polymer adhesive membrane on the surface of soil column.

When the surface of the soil column was consolidated by the ternary blend adhesive, the surface soil and the adhesive together formed a strong layer, as shown in Fig. 4a, b. When the ternary blend adhesive was dried on the surface of the soil column sample, a hard shell formed on the surface of the soil column, which made the consolidated soil column have a certain strength, as shown in Fig. 4b, c. The structure had high strength and good moisture retention, which secured the integrity of soil columns and the breathing of plant roots.

**Figure 5:**
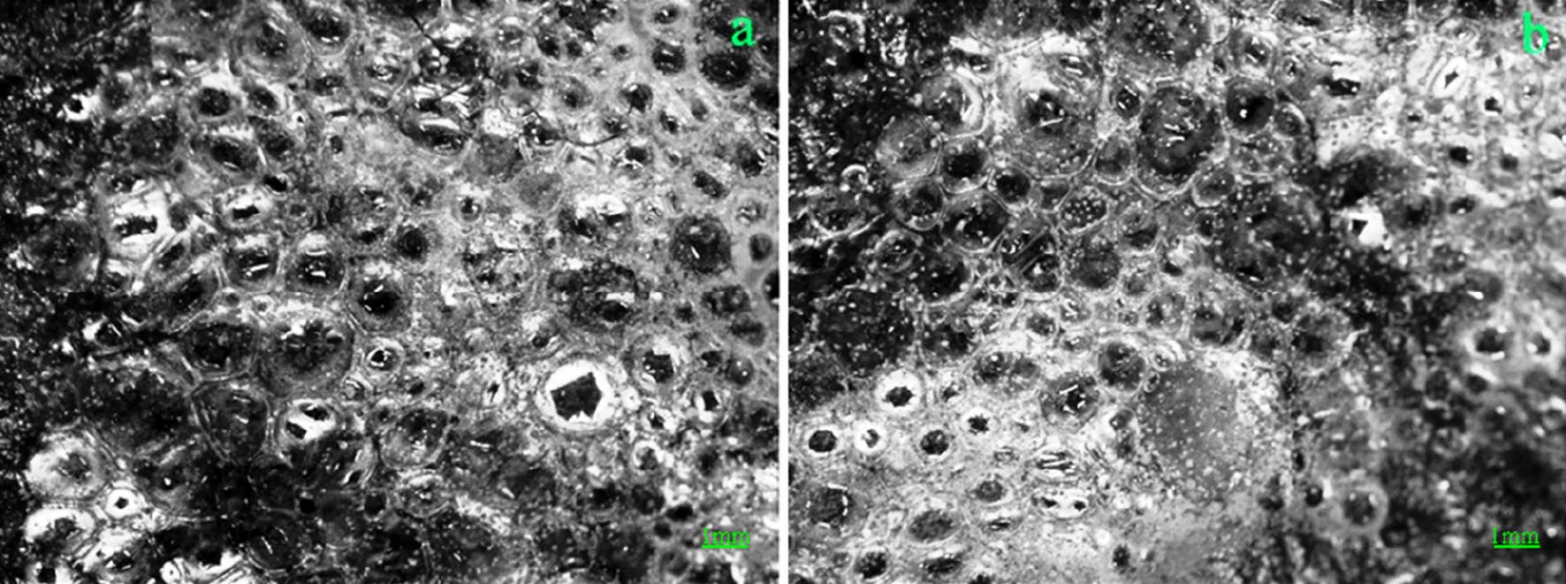
Cemented surface morphology of consolidated soil balls.

Then the surface morphology of consolidated soil cementation was shown in Fig. 5a, b. It was found that the cementing film formed on the surface of soil column was a network membrane structure formed by the connection of bubbles, as shown in Fig. 5a. The structure is stable and the membrane strength is high. The interior of each bubble wrapped soil particles and forms a convex membrane on the surface of the soil particles. The convex membrane of these bubbles adhered closely to the soil particles on the surface of the soil column, so that the soil on the surface of the soil column and the adhesive membrane formed a whole, so the surface of the soil column consolidated with the adhesive membrane had a certain intensity, as shown in Figs. 4c and 5b. The structure was stable and had some strength which would be tested later. The most important thing was the pores on the surface of cemented membrane, which were permeable for water and breathable for the roots.

### Compressive strength of consolidated soil balls

**Figure 6:**
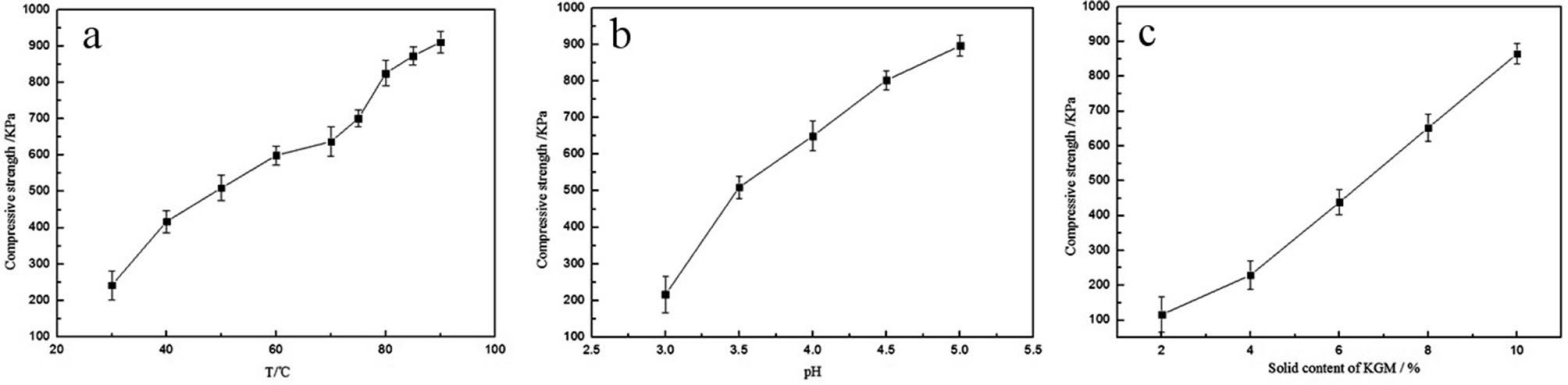
Effect of blending conditions on compressive strength of consolidated soil columns. (**a**) General conditions: Stirring time-40 min; Content of KGM, CA, PVA = 4.0%, 4.0%, 5.0%; pH of glue-4.5; pH of the Cinnamon soil-8.5; soil particle size-2 mm; (**b**) General conditions: Stirring time-40 min; T-85℃; Content of KGM, CA, PVA = 4.0%, 4.0%, 5.0%; pH of the Cinnamon soil-8.5; soil particle size-2 mm; (**c**) General conditions: Stirring time-40 min; T-85℃;KGM:CA = 1:1; Content of PVA-5.0%; pH of glue-4.5, pH of the Cinnamon soil-8.5; soil particle size-2 mm.

**Figure 7:**
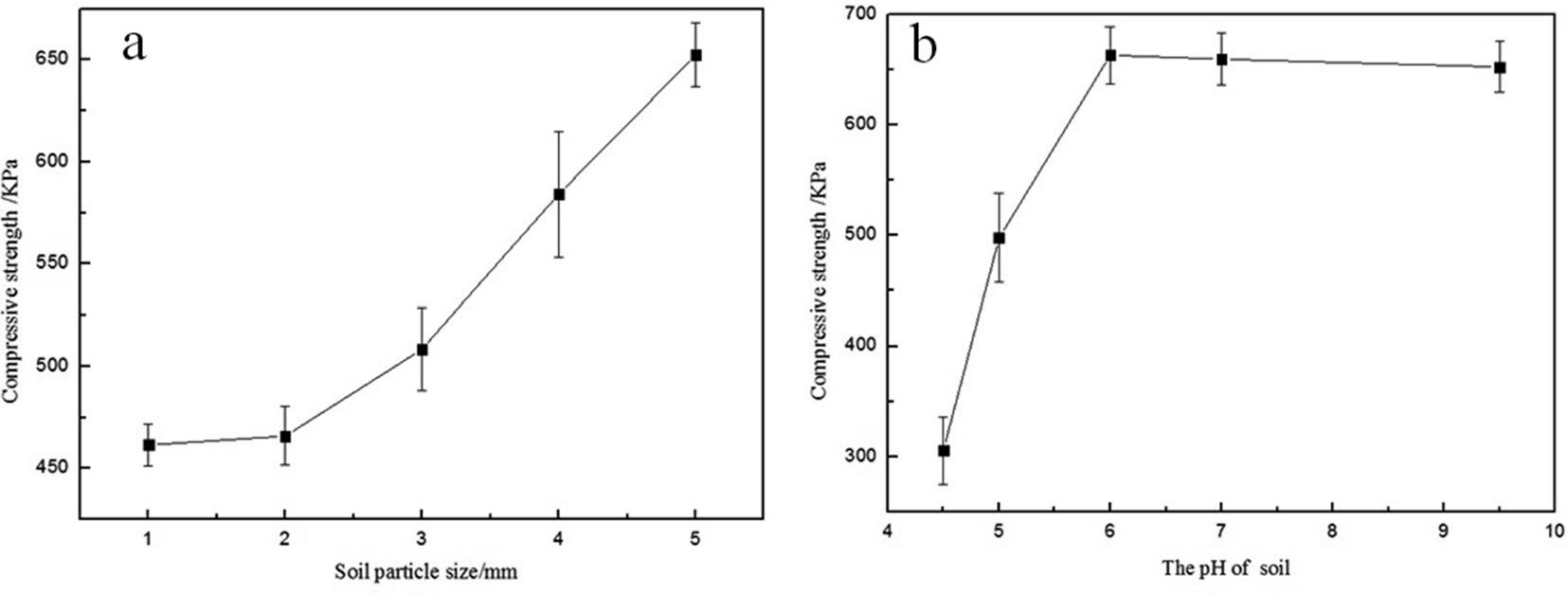
Effect of soil particle size and soil pH on compressive strength of consolidated soil column. (**a**) General conditions: Stirring time-40 min; T-85℃; Content of KGM, CA, PVA = 4.0%, 4.0%, 5.0%; pH of glue-4.5, pH of the Cinnamon soil-8.5; (**b**) general conditions: Stirring time-40 min; T-85℃; Content of KGM, CA, PVA = 4.0%, 4.0%, 5.0%; pH of glue-4.5, soil particle size-2 mm.

It was found that the blending temperature, pH of the glue liquid, solid content of the glue liquid, particle size of soil and pH of the soil had certain effects on the compressive properties of the consolidated soil balls, as show in Figs. 6 and 7.

The blending temperature had a great influence on the compressive properties of the consolidation soil ball. With the increase of the blending temperature, the compressive strength of the consolidated soil ball gradually increased, as shown in Fig. 6a. The pH of the liquid had a certain effect on the consolidation of soil consolidation agent. With the increase of pH, the compressive strength of the consolidated soil ball increased. In strong acidic environment, the intermolecular interaction of the blend polymer was weak and the adhesion was not strong enough. With the weakening of acidity, the compressive strength of consolidated soil columns increased gradually. The compressive strength of the consolidated soil column was 649 KPa at pH of 4.0, and 873 KPa at pH 4.5, as shown in Fig. 6b. The solid content of KGM and CA had certain effect on the compressive strength of consolidation soil balls. With the increase of the solid content of KGM in the ternary blend adhesive, the compressive strength of the soil balls also increased. The compressive strength of the consolidated soil column was 229 KPa at 4.0% of the KGM solid content, and 438 KPa at 6.0% of the KGM solid content, as shown in Fig. 6c. It can be seen that the effect of glue solid content on the compressive strength of soil columns was smaller than that of pH (Fig. 6b, c).

Furthermore, the polymer adhesive will be used to consolidate soil balls, so that it is needed to consider the influence of soil particles and pH of the soil. In the present study it was found that soil particle size also had a certain effect on the compressive strength of consolidated soil columns. With the increase of soil particle size, the compressive strength of consolidated soil columns also increased, as shown in Fig. 7a. When the soil particle size was small, the ternary blend adhesive could only penetrate into the surface layer of the soil column 1–2 mm deep, so that a thin film formed on the surface of the soil column. When the thin film was dried, parts of the film fell off from the surface of the soil column, resulting in the lower compressive strength of the consolidated soil column. With the increase of soil particle size, the ternary blend adhesive penetrated into the soil column deeper, and the depth reached more than 5 mm. The dried outer layer formed a thick shell, so that the compressive strength of the consolidated soil column increased. The pH of soil also had a great influence on the compressive strength of consolidated soil columns. With the increase of soil alkalinity, the compressive strength of consolidated soil balls first increased and then tended to be stable, as shown in Fig. 7b.

### Transportation test of consolidated soil column

**Figure 8:**
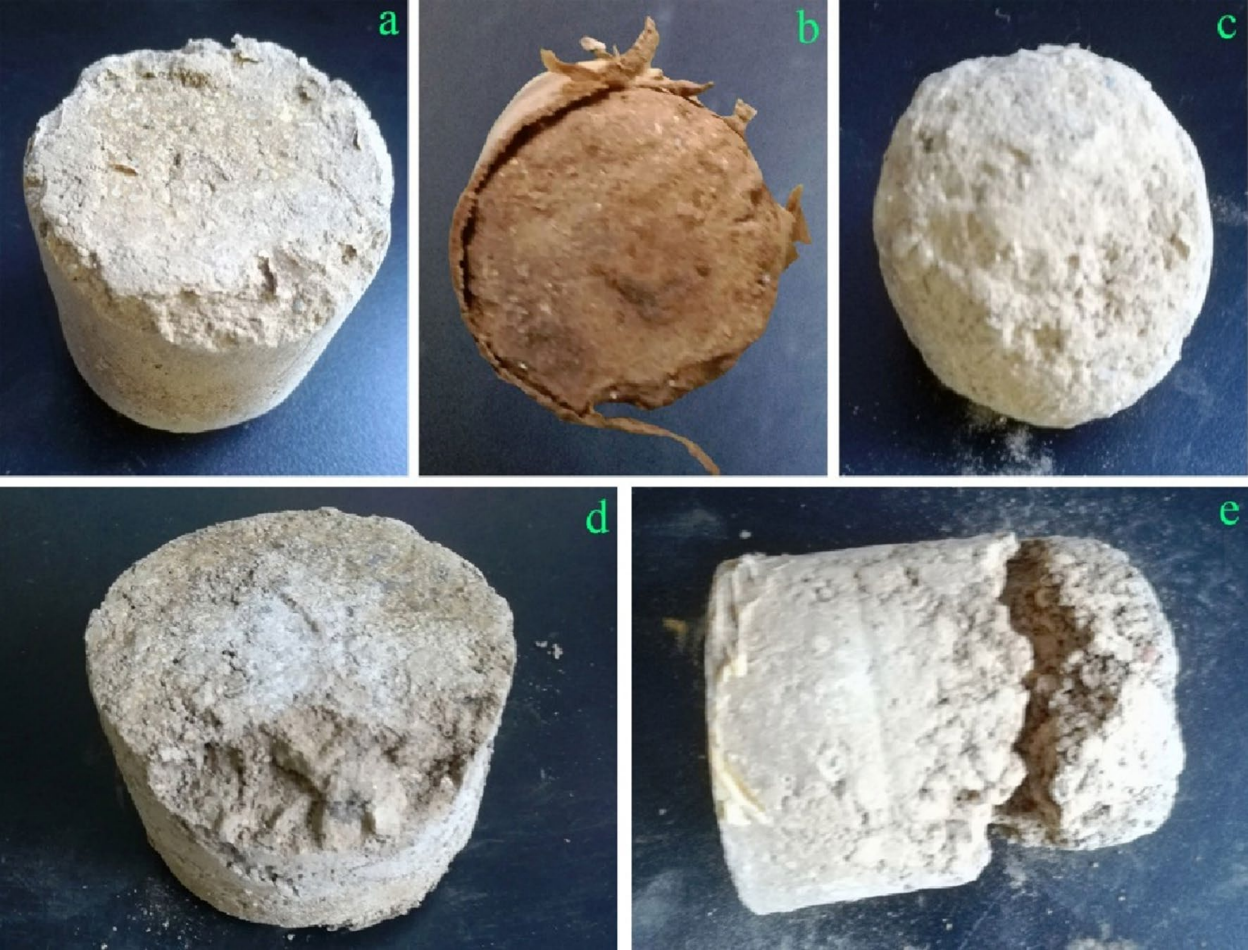
Typical soil column samples a-e of vertical vibration test.

The consolidated seedling root balls should be strong enough to endure seedling transportation from the nursery field to new growth sites such as the avenues, streets and parks. The group of tests includes freeway truck vibration test, combined wheeled vehicle vibration test and impact test. According to the experimental conditions (Table S1 in the Supporting Information), the simulated expressway mileage reached 1600 km. The test results showed that 35 soil columns consolidated by solid consolidation agent had no wear and tear, and none of them was broken during the vibration test of simulated freeway truck transportation. Transportation vibration tests of the three- or four-grade highways were carried out according to the vibration test conditions of combined wheeled vehicles (Table S2 in the Supporting Information). The transport mileage of the three- or four-grade highways simulated by the tests reached 400 km. In the transverse and longitudinal vibration tests of combined wheeled vehicle, these 35 soil columns consolidated with the solid consolidation agent were not worn or broken. But only in the vertical vibration test, the soil columns had varying degrees of wear. The rate of wear reached nearly 30%. The wear degree of soil column samples was different corresponding to the different preparation conditions and test positions (Table 1; Fig. 8).The general wear was concentrated on the edges and upper and lower parts of the soil column (Fig. 8a). The dry and wet state of the soil columns resulted in different wear (Fig. 8a,b). In the process of vertical oscillation, the close contact of soil columns leaded to severe wear of the circumference of soil column (Fig. 8c). Only two soil columns located next to the wall of the wooden box were fragmented (Fig. 8d,e). Therefore, the location of soil columns was critical in the vertical vibration test of combined wheeled vehicle. The impact test mainly simulated the sudden drop of the soil columns in the transportation, and the turning upside down and dropping of soil columns at passing through pits of the road during transportation. In three-axial impact tests, all the soil columns consolidated with polymer adhesive were not damaged, indicating that the soil columns consolidated with blend glue had stronger impact resistance.

**Table 1:** The experimental conditions of typical soil column samples a–e.

The experimental condition	Sample a	Sample b	Sample c	Sample d	Sample e
Type of soil column	Type2	Type1	Type1	Type2	Type1
Content of KGM (%)	5%	4.0	4.0	4.0	4.0
Content of CA (%)	5%	4.0	4.0	4.0	4.0
Content of PVA (%)	5%	4.0	1%	5.0	1%
pH of glue	3.5	5.0	4.0	4.9	4.3
T (℃)	85	85	25	85	85
pH of the soil	8.5	8.5	8.5	8.5	5.0
Soil particle size (mm)	2	5	2	Undisturbed soil	2
Test position in the box	The edge of upper layer	The edge of lower layer	The edge of lower layer	The edge of lower layer	The edge of lower layer

### Preliminary application of polymer as soil consolidation agent during tree transplanting

**Figure 9:**
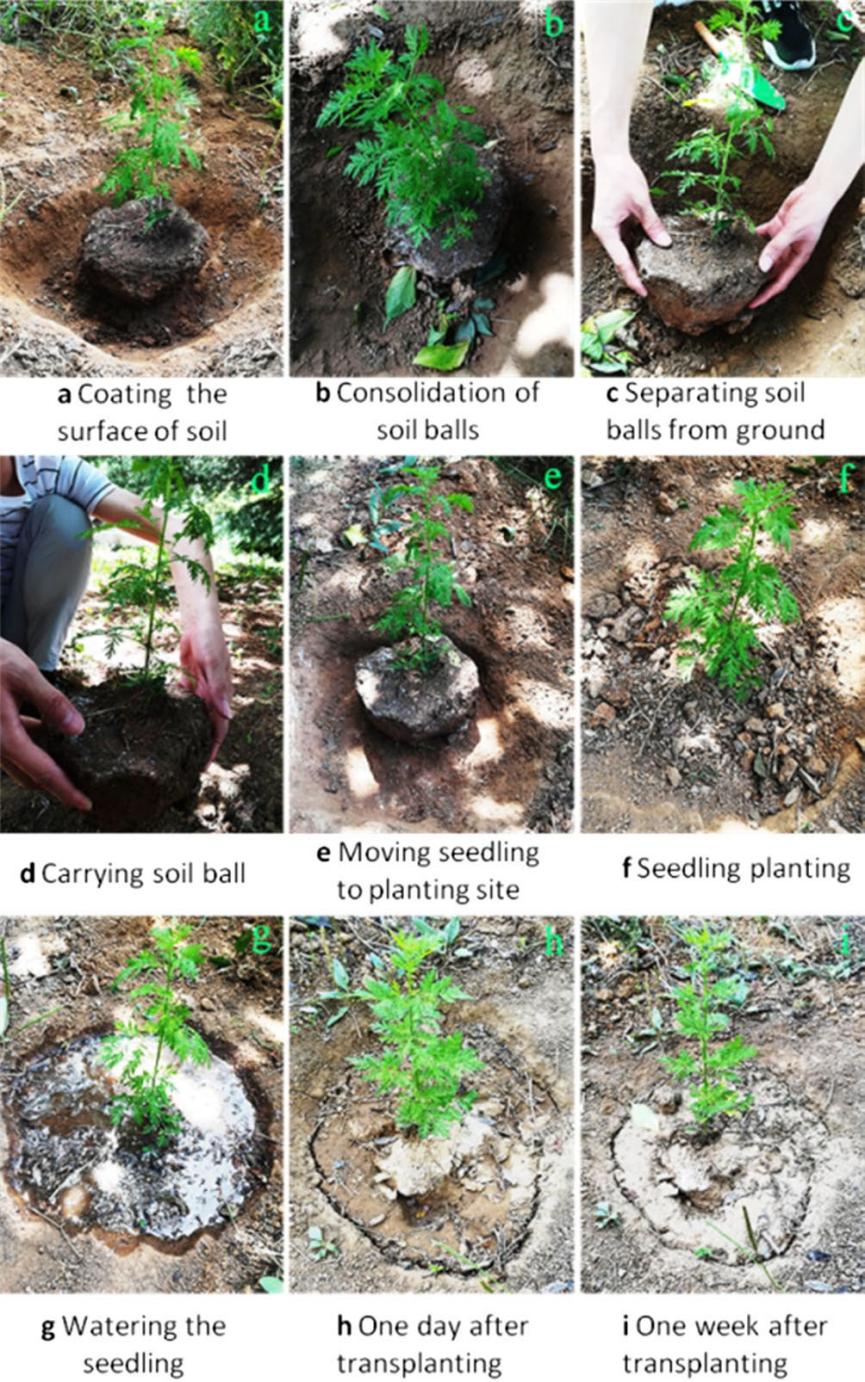
Process of transplanting sierra salvia with soil consolidation agent.

**Figure 10:**
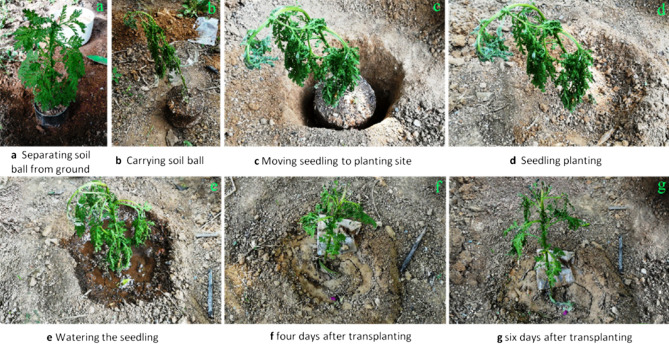
Transplanting sierra salvia with soil consolidation agent.

To detect the adhesive effect of this polymer as the soil consolidation agent, three kinds of plants, sierra salvia (*Artemisia brachyloba*), Japanese Spindle (*Euonymus japonicus*) and *Juniperus sabina* ‘Tamaricifolia’ were selected to carry out the transplanting experiment. Firstly, perennial herb sierra salvia was chosen as plant materials, and transplanting experiments were carried out twice separately. In transplanting case 1, sierra salvia with 5 mm at diameter at breast height (DBH) was used as the main object of transplanting. According to the requirement of transplanting, a circle with a diameter of 10–15 cm was drawn around the root with the stem as the center. Removing the soil surrounding the root ball shaped the root ball to a cone (top larger and bottom smaller), as shown in Fig. 9a. Then the polymer adhesive was spread on the upper surface and the side surface of the root ball. The root ball coated with the adhesive solidified in the outdoor environment for about a day, forming a hard shell on the surface of the ball, as shown in Fig. 9b. At this time, the root ball of sierra salvia was removed and directly transported to the new planting site, as shown in Fig. 9c–e. Then, according to the requirements of seedling planting, the seedling was planted and watered, as shown in Fig. 9f–g. On the next day the seedling grew well, as shown in Fig. 9h. After one week of transplanting, sierra salvia was completely adapted to the new environment, only a few yellow leaves appeared near the root (Fig. 9i). Within the next month, these yellow leaves slowly fell off and fresh green leaves gradually grew. Subsequent follow-up observation showed that the transplanted sierra salvia grew well. It indicates that the weak acidity of the soil consolidation agent not affected the growth of seedlings after seedling transplanting, as shown in Fig. 9. In transplanting case 2, sierra salvia was transplanted by harsh transplanting method to investigate whether soil consolidation agent could retain water in the root. After lifting of sierra salvia with the mould, the surface of soil ball around the root was spread with the polymer adhesive, as shown in Fig. 10a,b. After 3 days of sun exposure, sierra salvia was found to be wilted obviously (Fig. 10b). Then the sierra salvia was transplanted to the planting site, and the procedure of filling and watering was completed according to the requirements of transplanting, as shown in Fig. 10c–e. After follow-up observation, it was found that sierra salvia gradually and successfully survived, as shown in Fig. 10f,g. This indicates that the root ball prepared with soil consolidation agent had certain water retention to the root of sierra salvia.

**Figure 11:**
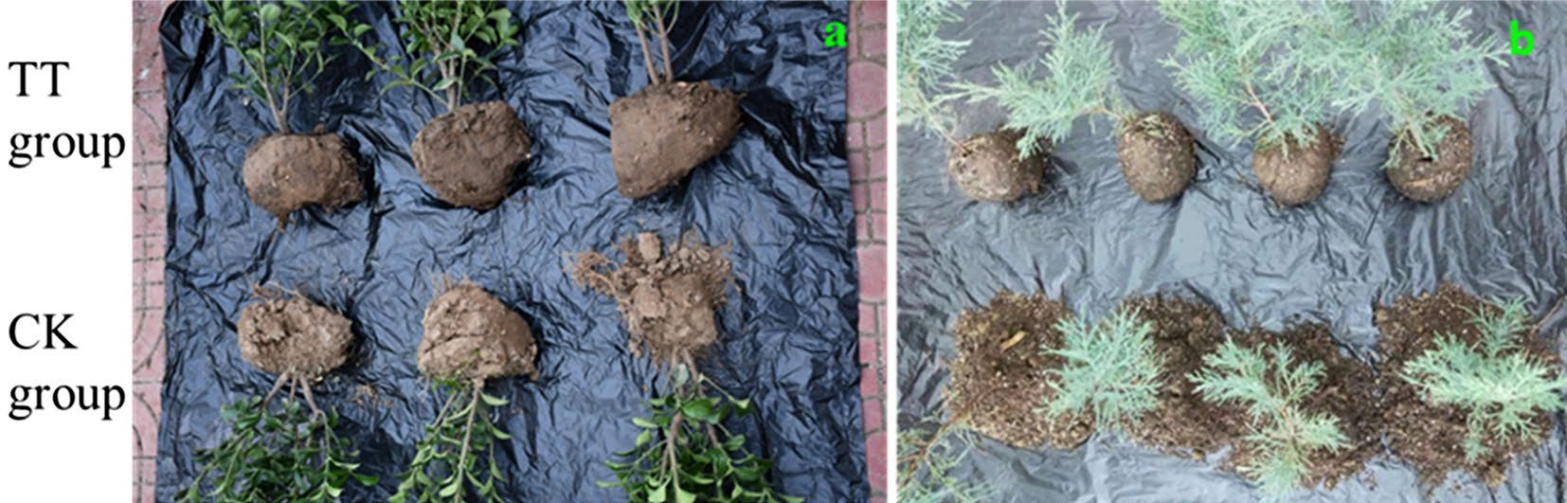
Application of ternary blend adhesive to Japanese Spindle (*Euonymus japonicus*) (**a**) and *Juniperus sabina* ‘Tamaricifolia’ (**b**).

To further determine the practical effects of the polymer and influence on tree, Japanese Spindle (*Euonymus japonicus*) and *Juniperus sabina* ‘Tamaricifolia’ were used to application materials, which belongs to broad-leaved plants and conifer plant, respectively. As shown in Fig. 11, soil balls treated with ternary blend adhesive (TT group) showed good adhesion within rhizosphoric soil, while the soil in the control group (CK group) which was not treated with ternary blend adhesive was loose, especially in the rhizosphoric soil of the *Juniperus sabina* ‘Tamaricifolia’ (Fig. 11b). These results suggested that the ternary blend adhesive had fine practical effect on tree transplanting.

**Figure 12:**
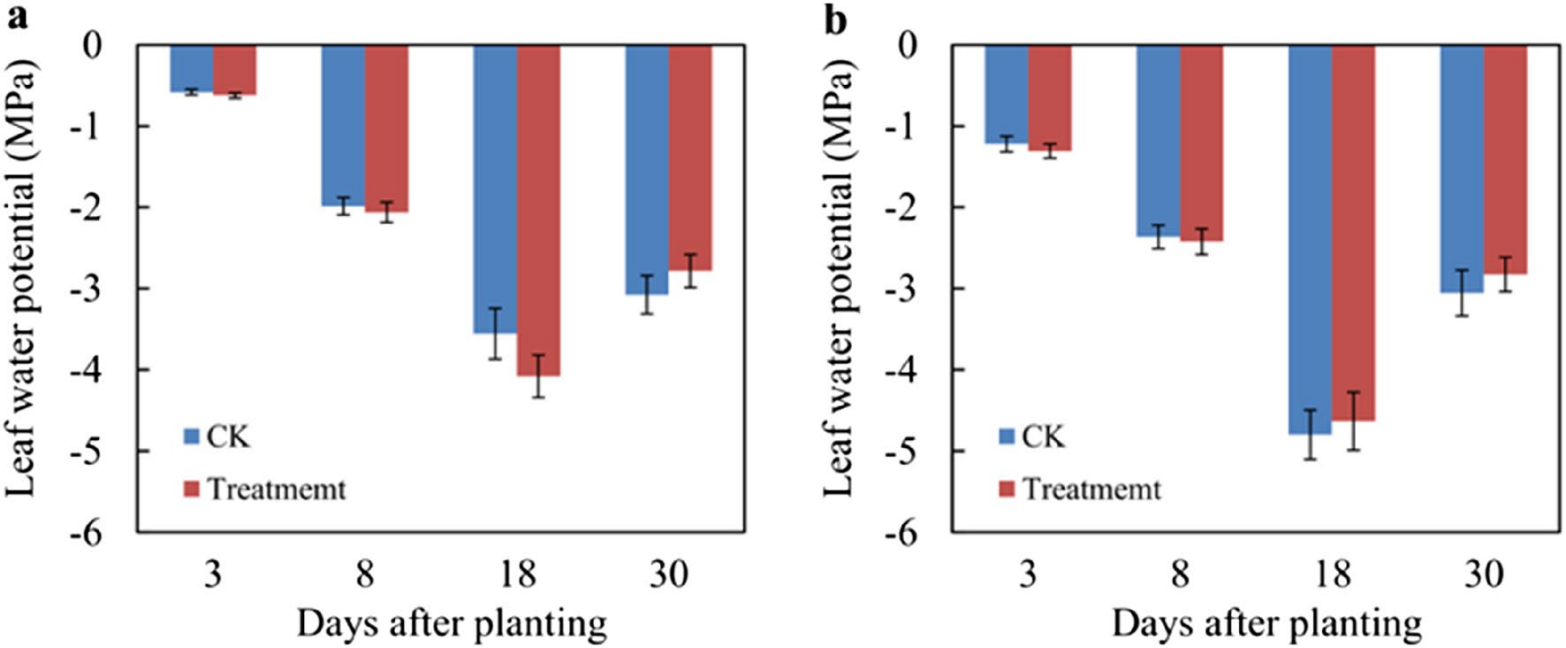
The Leaf water potential (ψ^leaf^) of Japanese Spindle (*Euonymus japonicus*) (**a**) and *Juniperus sabina* ‘Tamaricifolia’ (**b**). 30 plants were used to test the leaf water potential in each treatments. Water was given immediately after transplanting, 10 days and 25 days after transplanting to kept the plants survival during 30 days of observation period.

**Figure 13:**
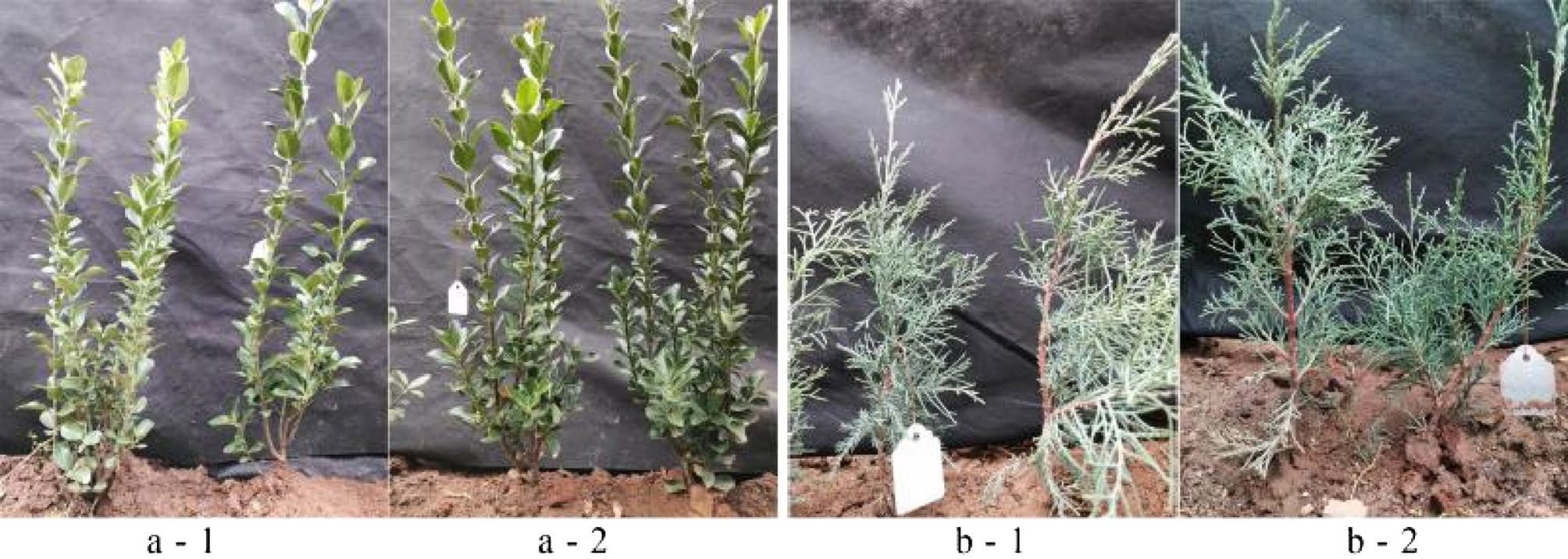
The leaf color and statement of Japanese Spindle (*Euonymus japonicus*) (**a**) and *Juniperus sabina* ‘Tamaricifolia’ (**b**) after 30 days of observation. Those with white hangtags were plant treated with polymer, while those without were controls.

Attempts to further evaluate the influence of ternary blend adhesive on plant survival, the plant statement was monitored under field conditions after transplanting during 30 days of observation period. Leaf water potential (ψleaf) and visible leaf color were used to reflect the survival of plant. As shown in Fig. 12a, there are no significant differences in ψleaf between CK and treatment both in Japanese Spindle (*Euonymus japonicus*) and *Juniperus sabina* ‘Tamaricifolia’, implying that the flow of water through the plant may be at similar levels. It is notable that, water was given immediately after transplanting, 10 days and 25 days after transplanting to kept the plants survival during 30 days of observation period. So the ψ^leaf^ at 8 and 18 days after planting was much lower than 3 days, because of water stress. Moreover, the ψleaf at 30 days declined along with the second rewatering at 25 days after transplanting, suggesting the survival of transplanted tree. As shown in Fig. 13, the leaf color was still green after 30 days of observation both in CK and Treatment, indicating that the tree was alive. Together with the visible color and leaf statement (Figs. 12, 13), it is concluded that both plants in CK and Treatment survived well, and no obviously adverse effect of ternary blend adhesive on plant survival. It should be noted that the correctly treated procedure is vital for plant survival. If the ternary blend adhesive was directly contacted with large root systems, the color of plant leaf and stem can turn yellow from green gradually (Fig. S1). But this changes could not affect the survival of transplanting plants. And this phenomenon has also appeared in the process of rejuvenation of transplanted sierra salvia. However, as the transplanted seedlings grow, the yellow leaves can gradually fall off and new leaves can grow, and eventually survive.

## Discussion

Transplanting trees with rhizospheric soil is a widely used way to facilitate tree survival in the process of landscaping and reforestation. It can reduce the damage to roots, especially young roots, and maintain the absorption capacity of roots to the greatest extent. Moreover, some plant roots and original soil rhizospheric microorganisms form special symbiotic interaction, and transplanting with soil can keep this original community as much as possible, which is conducive to the rapid adaptation of plants to the new environment and survive. Traditional way to prevent looseness of rhizospheric soil is forming soil balls around the roots with bags, boxes or rope wrapping, which is cumbersome, laborious and easy to break. It is necessary to exploit a new type of polymer as soil consolidation agent to facilitate tree transplanting. In consideration the safety of environment and transplanted trees, it must make sure that the polymer is degradable. In this study, the KGM/CA/PVA ternary blending soil consolidation agent was prepared by using KGM, CA, PVA as raw materials. We studied its characteristics and primary application in tree transplanting.

### Screening of preparation conditions of ternary blend adhesives

Consolidated soil balls need to bear a certain self-weight. The high the viscosity of adhesives, the greater the strength of consolidated soil balls would be. In the test of the influence of blending temperature on the viscosity of the ternary blend adhesive, it was seen that the higher the blending temperature, the higher the viscosity of the glue. Especially nearly 85 ℃, the viscosity of the glue increased rapidly. Therefore, it is reasonable to choose the ternary blend adhesive prepared at 85 ℃. The effect of pH on viscosity of ternary blend adhesives indicated that with the increase of the pH, a large number of intramolecular and intermolecular hydrogen bonds were formed among –NH_2_, –OH on the molecular chains of CA and –OH on the molecular chains of KGM. From this result the pH of the ternary blend adhesives should be chosen under acidic condition, say at pH lower than 5. With the increase of solid content of KGM, CA and PVA, the viscosity of blend glue increased linearly. For the practical application, a balance between the solid content and the viscosity should be chosen. The selection of blending temperature, pH value and solid content were the prerequisite of the following consolidation steps. By considering the viscosity and fluidity of the glue, the preparation conditions of ternary blend adhesive were as follows: blending temperature was 85 °C, the pH of the ternary blend adhesive was 4.5, the content of KGM was 4.0%, CA 4.0%, and PVA 5.0%.

### Temporarily network of KGM/CA/PVA ternary blend adhesive

**Figure 14:**
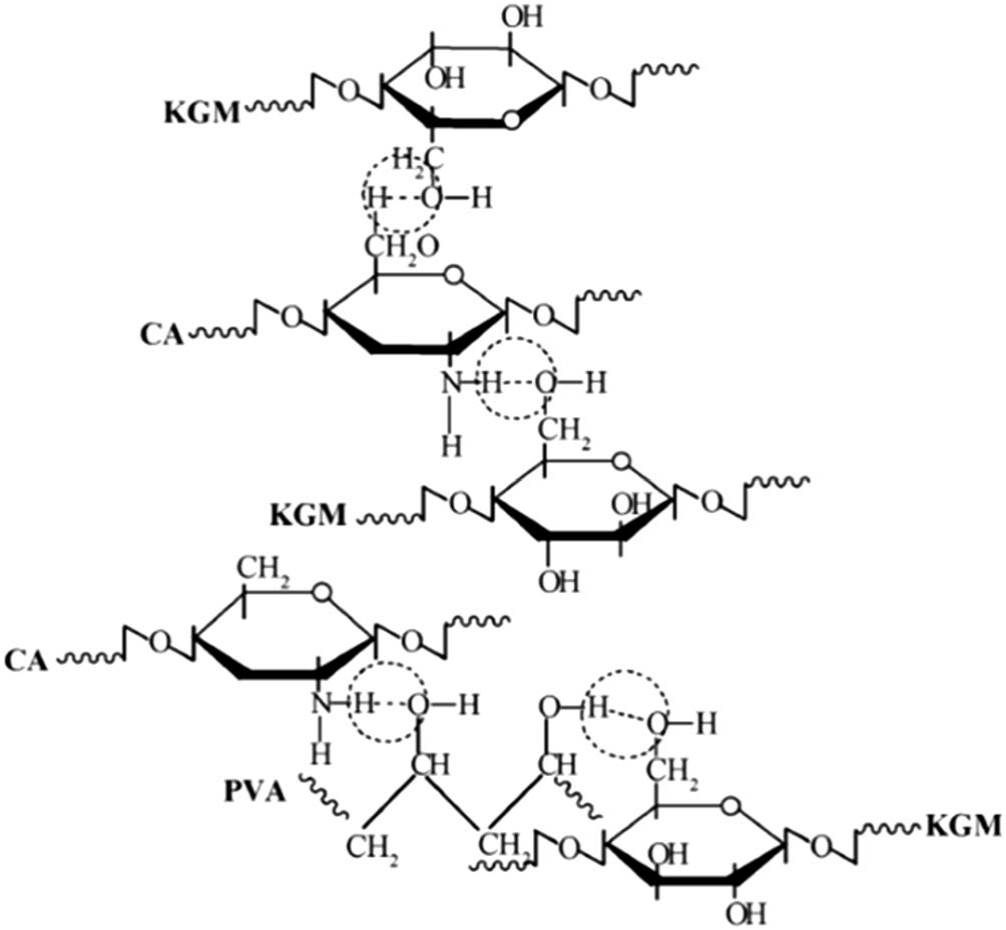
The interaction between KGM, CA and PVA.

FTIR analysis confirmed the interaction between KGM, CA and PVA, as shown by the dotted circles (Fig. 14). In KGM and CA molecules, there was not only the interaction between amino group and alcohol hydroxyl, but also there was a certain bond between hydroxyl groups, indicating the formation of hydrogen bonding between KGM and CA molecules. The chains of PVA contained a large number of hydroxyl groups, while KGM and CA chains contained a large number of hydroxyl groups and amino groups. There were hydrogen bonding interactions between KGM and PVA, and between CA and PVA. KGM, CA and PVA could form strong hydrogen bonds among each other, coupling with the interaction between KGM and CA molecular chains, so the molecular chains of KGM, CA and PVA interacted with each other to form a three-dimensional network structure after blending.

It can be seen that a three-dimensional network structure was formed temporarily by hydrogen bonding among the molecular chains of KGM, CA and PVA in the blend glue. This was beneficial to the formation of a network-like film on the surface of the soil ball to ensure certain compressive strength of the consolidated soil ball.

### Balance of moisture permeability and water retention of polymer blend membranes

In the process of seedling transportation, the seedling roots wrapped in earth balls need a certain water retention of the polymer blend membranes to ensure the water demand of the seedling roots. At the same time, after transplanting seedlings in new sites, the roots of seedlings need to absorb water from the soil of the planting site before the collapse and degradation of consolidated polymer blend membranes. Therefore, the polymer blend membranes also need to have certain moisture permeability. It is clear that the polymer blend membranes need to reach a balance between water permeability and water retention.

With the increase of blending temperature, the moisture permeability of polymer blend membranes decreased, while the viscosity of the ternary blend adhesive increased gradually. After transplanting seedlings, the absorption of water by the roots of seedlings is a slow process before the collapse of the polymer blend membrane. The polymer blend membrane should have a certain moisture permeability to meet the application requirements. The most important thing is to ensure the viscosity of the ternary blend adhesive. Therefore, it was reasonable to choose the preparation temperature of the ternary blend adhesive at 85 ℃. The solid content selection of ternary blend adhesive was also for a similar reason, so the solid content of the ternary blend adhesive was chosen as follows: the content of KGM was 4.0%, CA 4.0%, and PVA 5.0%. With the increase of pH, the moisture permeability of the polymer blend membranes decreased gradually, while the viscosity of the ternary blend adhesive increased. If the acidity of the ternary blend adhesive was too strong, it would not only be harmful to the growth of seedlings, but also affect the viscosity of the ternary blend adhesive. By considering comprehensively, it was reasonable to choose the pH value of ternary blend adhesive as 4.5.

### Consolidation of soil with polymer adhesives

**Figure 15:**
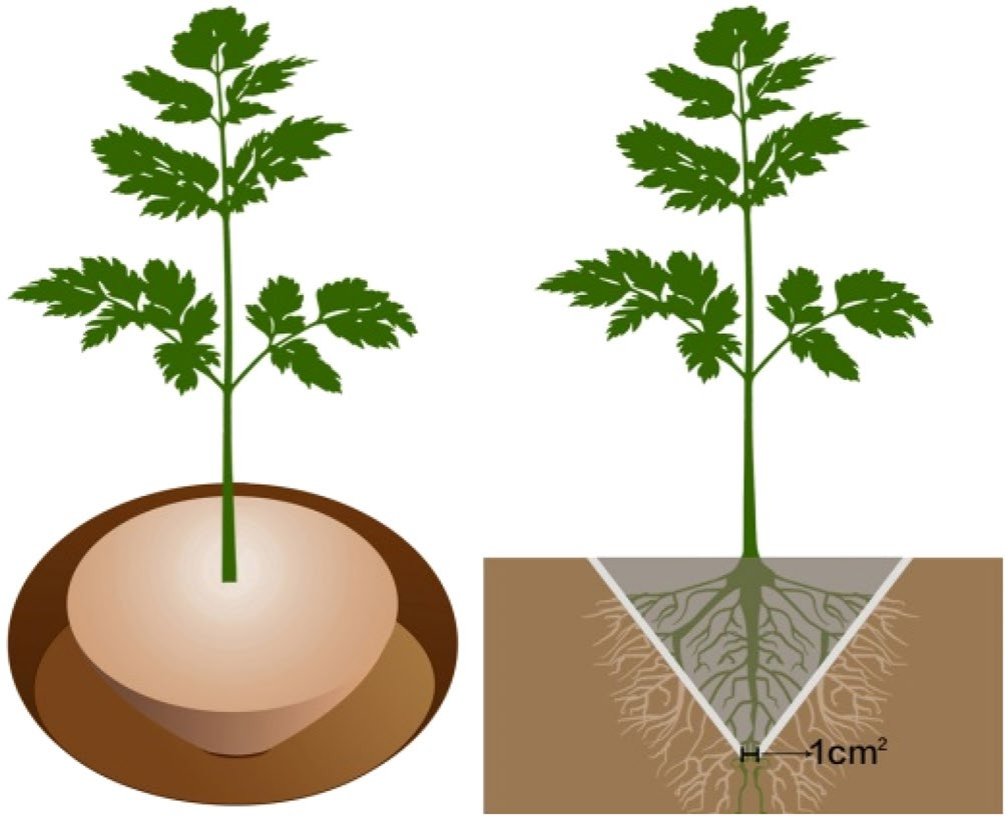
The model of conical soil ball.

When the ternary blend adhesive was dried on the surface of the soil column sample, a hard shell formed on the surface of the soil column, which made the consolidated soil column have a certain strength (Fig. 15). The compressive strength test showed that the compressive strength of soil column increased with the increase of temperature, pH, solid content of ternary blend adhesive and soil particle size. However, in the process of transplanting seedlings, the root of seedlings needed different amounts of water at different stages, so it was necessary to consider the mechanical properties, the permeability and water retention of polymer adhesive membranes at the same time. According to the current experimental results, it was reasonable to choose the preparation conditions of the blending temperature 85℃, the pH of ternary blend adhesive as 4.5, and the content of KGM as 4.0%, CA as 4.0% and PVA as 5.0%. It can be seen from the influence of soil pH on the compressive strength of consolidated soil column that the stronger the soil acidity was, the weaker the adhesion of the glue was to the soil, resulting in the lower compressive strength of the consolidated soil column. Thus it can be seen that the acidic blend glue is more suitable for the consolidation of alkaline and neutral soil.

**Figure 16:**
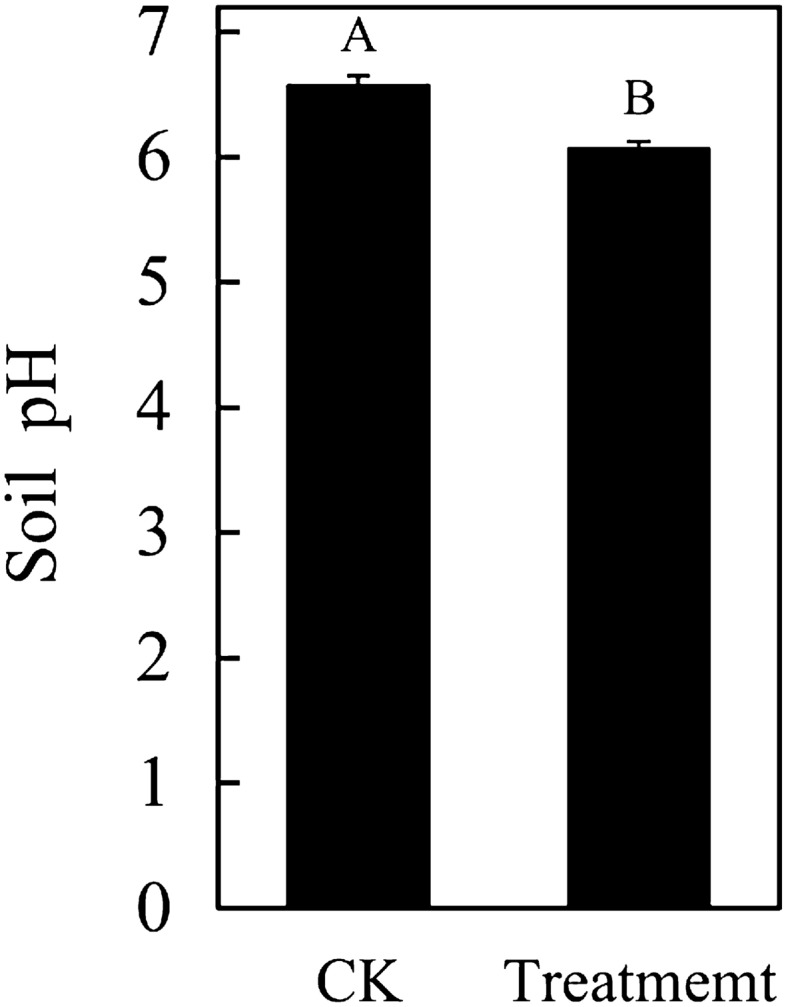
The pH of consolidated soil column.

Certain extents of soil pH values were necessary for plant growth and survival. From the effect of ternary blend adhesive on soil pH, it was showed that the application of ternary blend adhesive could reduce the soil pH values significantly, as shown in Fig. 16. However, the change was still in certain extents which plant can endure. The results confirmed again that ternary blend adhesive in our study might be more suitable for alkaline and neutral soil.

In summary, it is reasonable that the high enough viscosity with the temporary network of ternary adhesives led to the stronger consolidated soil columns. From the results of three kinds of vibration tests of the consolidated soil column, it can be seen that the consolidated soil column has a high enough strength to withstand the severe vibration and shock in road transportation. And under the preparation conditions of ternary blend adhesive (the blending temperature 85℃, the pH of ternary blend adhesive as 4.5, and the content of KGM as 4.0%, CA as 4.0% and PVA as 5.0%), the consolidated soil columns kept certain water vapor permeation.

### Influence of ternary blend adhesive on tree transplanting and plant survival

As a soil consolidation agent during tree transplanting, it is very important to possess good consolidation effect and keep the soil ball from loosening. It this study, we found that the ternary blend adhesive have fine practical effect on tree transplanting. As shown in Figs. 9, 10, 11, soil balls treated with ternary blend adhesive (TT group) showed good adhesion within rhizosphoric soil, while the soil in the control group (CK group) which was not treated with ternary blend adhesive was loose. There are three kinds of plants, sierra salvia (*Artemisia brachyloba*), Japanese Spindle (*Euonymus japonicus*) and *Juniperus sabina* ‘Tamaricifolia’, which represent perennial herb, broad-leaved tree and conifer tree, correspondingly. According to the transplanting experiments with the three kinds of plants, it is concluded that this ternary blend adhesive can be widely used during tree transplanting. What’s more, we further evaluate the influence of ternary blend adhesive on plant survival and growth, according to observation the leaf water potential (ψleaf) and visible leaf color during 30 days of observation period. As shown in Fig. 12, there are no significant differences in ψleaf between CK and treatment both in Japanese Spindle (*Euonymus japonicus*) and *Juniperus sabina* ‘Tamaricifolia’, implying that the flow of water through the plant may be at similar levels. It is notable that, the ψleaf at 8 and 18 days after planting was much lower than 3 days, because of water stress. However, the ψleaf at 30 days declined along with the second rewatering at 25 days after transplanting (Fig. 12), suggesting the survival of transplanted tree. Moreover, the leaf color was still green after 30 days of observation both in CK and Treatment (Fig. 13), indicating that the tree was alive. Above all, no obviously adverse effect of ternary blend adhesive on plant survival and short-term growth. It is notable that the correctly treated procedure is vital for plant survival and growth. If the ternary blend adhesive was directly contacted with large root systems, it may cause adverse effects on plants (Fig. S1). Of course, this adverse effect can be eliminated by extensive and multiple watering.

## Conclusions

In order to solve the problem that the soil ball is easy to be broken in the process of seedling transplanting, the ternary blending soil consolidation agent was prepared by using KGM, CA and PVA as raw materials in this paper. From the viscosity of the ternary blend adhesive, the structure of KGM/CA/PVA ternary blend membrane and the surface morphology compression resistance and vibration resistance of the consolidated soil ball, the rationality of the preparation conditions of the ternary blend adhesive (the blending temperature 85℃, the pH of ternary blend adhesive as 4.5, and the content of KGM as 4.0%, CA as 4.0% and PVA as 5.0%) was comprehensively verified. The KGM/CA/PVA ternary blend adhesive with the best performance was applied to the transplanting of sierra salvia, Japanese Spindle (*Euonymus japonicus*) and *Juniperus sabina* ‘Tamaricifolia’ and it was found that the application of soil consolidation agent can effectively solve the problem that the root ball of seedling is easily broken in the process of transplant. In addition, the observation results of the leaf water potential (ψ^leaf^) and visible leaf color of the seedlings indicated that the growth of the transplanted seedlings can not be affected as long as the ternary blend adhesive was avoided from contacting the roots of the transplanted seedlings during the adhesive coating spraying.

In this paper, a new type of soil consolidation agent was proposed to consolidate the soil ball at the root of transplanting seedlings to ensure the integrity of the soil ball in the process of transportation, which is a feasible scheme for new technology for seedling transplanting. In this paper, the research and development of environment-friendly degradable soil consolidation agent is the formation of new seedling transplanting technology. Therefore, the study of the new soil consolidation agent in this paper is of great significance to promote the formation of new seedling transplanting technology.

## Materials and methods

### Materials

The main raw material konjac flour (KGM, 200 g/bottle) was provided by Bozhou Baofeng biotechnology limited company. Chitosan (CA, chemical pure), polyvinyl alcohol (PVA, superior-grade pure), acetic acid (excellent-grade pure), sodium hydroxide (analytical purity) and other compounds were supplied by Sinopharm Chemical Reagent Co., Ltd. of China.

### Experimental methods

#### Principal consideration and preparation of polymer-type soil consolidation agent

The choice of polymers for consolidating soil should meet some criteria. Firstly, the preparation of soil consolidation agent should be environmentally friendly, non-toxic and degradable, so konjac glucomannan (KGM) and chitosan (CA) were chosen as the main components. Secondly, the polymer-type consolidation agent should have certain fluidity for easily spreading over the soil ball and penetrating into the soil cracks, and have a viscosity high enough to adhere the soil together tightly. Thirdly, consolidated soil balls wrapping the roots of seedlings should let the roots of seedlings to breathe, so the polymer film formed on the surface of root balls should have a certain permeability. Fourthly, in the transportation, the seedlings to be transplanted can only absorb water from the soil ball through the root, so the polymer film formed on the surface of the soil ball should have certain water retention.

Referring to previous experimental methods^32^, a binary polymer glue was prepared by blending KGM and CA as the main components. Then the tackifier polyvinyl alcohol was added into the KGM/CA binary blend system. After the blending reaction, a KGM/CA/PVA ternary blend was obtained. In the preparation of the KGM/CA binary blend and the KGM/CA/PVA ternary blend, three important factors were investigated: the blending temperature, the pH of these liquid blends, and the solid content of the liquid. Based on the results, a fixed formulation of the polymer adhesive was used for the study of strength of root balls, seedling transportation and transplantation.

### Infrared testing method for ternary blend adhesive

KGM and CA were prepared by pressing method, while PVA and KGM/CA/PVA ternary blend adhesive were prepared by film method. Then an IR-960 Fourier Transform Infrared (FTIR) Spectrometer was used to test KGM, CA, ternary blend films and PVA film, respectively. The wavelength range of the test was 4000–450 cm^−1^.

### Preparation of test films of polymer adhesive

Quantitatively KGM/CA or KGM/CA/PVA glue was poured into the middle of the operating table of an automatic film-coating machine for making films. After drying, these uniform films were used for further measurements of permeability and water retention. Then turned on the instrument switch. With the scraper moving back and forth, a layer of about 0.3 mm thick smooth film was successfully prepared. After a day of drying, the film was dried.

### Moisture permeability test

According to GB/T 1037–1988, water vapor permeability of polymer adhesive membranes was measured by a moisture permeability tester.

The calculation formula is as follows:

1 $$WVT=\frac{24\cdot\Delta m}{A\cdot t}$$

where WVT—Water vapor permeance, g/m^2^·24 h; t—interval time after stabilization of mass increment, h; Δm—Mass increment in t time; A—the permeable vapor area of the membrane sample was 0.0061m^2^.

### Preparation and tests of soil column samples

After the soil was thoroughly dried in the oven, 400 g dried soil was weighed and mixed with 100 g pure water and stirred evenly. Then the wet soil was made into two different types of soil columns with a ring knife as mold (model 1: mold diameter 50 mm, height 50 mm; model 2: mold diameter 100 mm, height 64 mm). The type1 soil column prepared by mould 1 needed 100 g of moist soil, while the type 2 soil column prepared by mould 2 needed 400 g of moist soil. After demoulding, the side surface of columns was coated with the ternary blend adhesive, and, the upper and lower surfaces were coated as well at room temperature. Each type 1 soil column required 10 g of ternary blend adhesive, while each type 2 soil column required 40 g of ternary blend adhesive. In rainless days, the surface glue consolidated these soil columns after drying for 1–2 days outdoor.

Then the surface morphology of consolidated soil cementation was observed with an OLYMPUS DP12 microscope. Then the consolidated soil columns were tested of their compressive strengths and anti-vibration performance during transportation. The compressive strength tests of consolidated soil columns were carried out on a Mechanical testing machine INSTRON 5582. According to GJB150.16A-2009 Military Equipment Laboratory Environmental Test Method—Vibration Test, the anti-vibration performance of consolidated soil columns during simulated transportation was tested. The transportation tests of consolidated soil columns were conducted by the BT900 hydrostatic horizontal sliding platform and the electric shaking-table experiment system. The group of tests includes freeway truck vibration test, combined wheeled vehicle vibration test and impact test. The test conditions of truck transportation on expressways, combined wheeled vehicle transportation and impact test are shown in Table S1–S3 in the Supporting Information.

Totally 35 soil columns consolidated with solid consolidation agent were closely put in two layers in a wooden box, as shown in Fig. S2a–c. The gaps between columns were filled with soft plastic foam to keep these columns from moving. Then, the wooden box with soil columns was placed onboard the BT900 hydrostatic horizontal sliding platform (Fig. S2d) for the vibration test on Expressway transportation. The whole test included tests in two different directions: longitudinal and transverse. In addition, the third direction test, i.e. the vertical test, was conducted on the electric shaking-table experiment system (Fig. S2e). The vibration test of combined wheeled vehicle and impact test was also completed by two instruments of BT900 hydrostatic horizontal slide platform (Fig. S2d) and the electric shaking-table experiment system (Fig. S2e).

### The determination of soil pH after application of polymer

The soil solution is prepared by adding distilled water into the soil ball in a vessel with the ratio of Water∶soil = 1.25∶1. After shaking ten minites, the vessel is then allowed to stand for 24 h. The soil pH was determination by Mettler Toledo with InLab@ Expert Pro-ISM electrode.

### Transplantation of seedlings

The whole transplantation of seedlings with the polymer-type consolidation agent was tested with the growth of an outdoor 5 mm DBH (diameter at breast height) sierra salvia as the main object. First, with the sierra salvia trunk as the center a circle of diameter about 10 cm-15 cm was drawn. Then the soil on the outer side of the circle column was cleaned from top to bottom until a conical (top larger and bottom smaller) soil ball was formed at the root of the sierra salvia, as shown in Fig. 15. The contact surface between the bottom of the conical soil ball and the earth was nearly 1cm^2^. Subsequently, the polymer glue was then spread on both the upper surface and the side surface of the conical soil ball. After 1-day drying, the glue on the surface of the soil ball was consolidated and formed a hard shell outside. At this moment the soil ball was directly transported to the new planting site for transplantation. According to the planting requirements of the seedling, filled the soil around the soil ball at the seedling root firmly, to avoid the pores around the root system, and made well the watering cofferdam.

Watering after transplanting was essential and done normally, but there was no exact procedure for the quantity and timing. Factors such as soil texture, temperature, wind and the size of the tree or seedling itself made the watering flexible. To prevent the root from rotting, kept the roots moist but not soggy. According to the experience, the newly planted seedlings should be watered three times consecutively to conserve water, and should be watered slowly and mildly with small amount of water, so as to help the roots of seedlings absorb sufficient water and avoid soaking in water. Depending on the time, place and weather of transplantation of the sierra salvia and the experience of seedling transplanting, the amount of water for sierra salvia was estimated and strictly enforced. Then the growth of sierra salvia was observed daily and regularly.

### Application and influence of polymer on plant seedlings

Another two kinds of plants, Euonymus japonicus and Juniperus sabina ‘Tamaricifolia’ were used as plant materials. In each kind of plant, 60 plants were divided into two groups: one group treated with polymers was named as Treatment while the other without polymers was named as CK. The specific application procedure was as follows: First, around the plant stem, rhizospheric soil was formed as a soil ball as shown in Fig. 15, with the diameter 1 ~ 1.5 folds of the crown. Then the polymer glue was spread on the surface of soil ball, be careful not to contact the root system. After 1-day drying, when the glue on the surface of the soil ball was consolidated and formed a hard shell outside, the treated plants should be transported to planting destination for transplantation. For transplanting, CK and Treatment plants were random arranged in one plot. Water was given immediately after transplanting, 10 days and 25 days after transplanting to kept the plants survival during 30 days of observation period.

To evaluate wether the polymer affect the survial of plants, leaf water potential and leaf color was observed during 30 days of observation period. Leaf water potential was detected by PSYPRO water potential system connected with C-52 sample chamber. System parameter modification was as fellowing: Psy cool = 30 s, Plat = 10 s, Av = 0.2 s, Scan = 10 s. The detect time point was at 12:00–14:00 for Euonymus japonicus, while it was at 14:00–16:00 for Juniperus sabina ‘Tamaricifolia’.

## Supplementary Information

Supplementary Information.
