# Supplementary material for: Study on a new type of environment-friendly polymer and its preliminary application as soil consolidation agent during tree transplanting
Source: Sci Rep. 2021 Mar 10;11:5575. doi: 10.1038/s41598-021-83594-2 (PMC7946934; doi:10.1038/s41598-021-83594-2)
Supplement: Supplementary file 1 — Supplementary Information. [file 41598_2021_83594_MOESM1_ESM.docx]

**Supplementary Information**

**Study on a new type of environment-friendly polymer and its preliminary application as soil consolidation agent during tree transplanting**

**Shaoli Wang^1^, Donglu Wei^2^, Xuping Yang^1^, Shengju Song^3^, Lifang Sun^1^, Xuebing Xin^1^, Guangshun Zheng^1^, Ran Wang^1^, LiLi Liu^2^, Jingshuang Sun^1^,Haixia Wang^1^, Fuling Lv^1^, Wenjuan Mo^1^**, **Hong Wang^2^, Chaoxing Luo^2^, Zhengqi Xiong^2^** **and Shaobo Wang^4^，Shaofeng Li^1*^, Yongxiu Xia^1*^**

^1^Experimental Center of Forestry in North China, Chinese Academy of Forestry, Beijing, 102300, China

^2^College of material and chemical engineering, Heilongjiang Institute of technology, Harbin, 150050, China

^3^R & D Center, China Academy of Launch Vehicle Technology, Beijing, 100076, China

^4^Foreign language teaching and research press, Beijing, 100089, China

*Corresponding author: Yongxiu Xia Email: [qjdhtynite@163.com](mailto:qjdhtynite@163.com);

Shaofeng Li Email: [lishaof2009@163.com](mailto:lishaof2009@163.com)

| Frequency of turning point（Hz） | Power spectrum density（g^2^/Hz） | Root mean square acceleration | Test time | Test directions |
| --- | --- | --- | --- | --- |
| 2 | 0.01500 | 1.10g_rms_ | 30min/direction | Longitudinal direction  Transverse direction  Vertical direction |
| 40 | 0.01500 |  |  |  |
| 500 | 000015 |  |  |  |

**Table S1**. Vibration test conditions for truck transportation on Expressways

**Remarks**: Random vibration test is generally simulated in transport environment, and the scanning frequency is relatively uniform. The input signal of random vibration is power spectrum density (PSD). Therefore, the following five paragraphs are the explanation of the random vibration test.

Random vibration refers to a kind of vibration whose waveform is chaotic, whose instantaneous value can not be predetermined at any given time in the future, and whose wave property can not show a certain regularity of vibration with the time transformation, so its regularity can not be explained by deterministic function.

Random vibration is also composed of sinusoidal vibration, while the frequencies of these sinusoidal vibration are not discrete, and continuously distributed in a certain range. The amplitude and displacement of each sinusoidal vibration will change unpredictably with time, and expressed by the mean, mean square, probability density function and power spectrum density of the random vibration signal at a certain time.

Power spectrum density (PSD) is an important parameter in random vibration. It can be understood as the magnitude of vibration energy per unit of frequency. The larger the value, the larger the amplitude of corresponding frequency band was. It can obviously sense the increase of the amplitude when the PSD value of the lowest frequency is increased in the experiment. There are two units of PSD: g^2^/Hz, (m^2^/Hz)^2^/Hz.

Frequency is another important parameter of random vibration. Its unit is Hz. The selection of frequency is generally related to the practical application range.

The third important parameter of random vibration is the root mean square acceleration g_rms_. It is calculated by calculating the area under the spectrum curve and then opening the root number. If the PSD is a flat line, the formula is g_rms_=$\sqrt{\mathrm{wf}}$, where w is the PSD value and f is the frequency value, whose value is equal to the highest frequency minus the lowest frequency. The g_rms_ value is similar to the g value of sinusoidal vibration. It is related to the maximum thrust of the equipment and is an important parameter for selecting the equipment.

| Vibration condition of combined wheeled vehicle | | | | | |
| --- | --- | --- | --- | --- | --- |
| Vertical direction | | Transverse directions | | Longitudinal direction | |
| Hz | g^2^/Hz | Hz | g^2^/Hz | Hz | g^2^/Hz |
| 5 | 0.2366 | 5 | 0.1344 | 5 | 0.0593 |
| 8 | 0.6889 | 7 | 0.1075 | 8 | 0.0499 |
| 12 | 0.0507 | 8 | 0.1279 | 15 | 0.0255 |
| 21 | 0.0202 | 14 | 0.0366 | 16 | 0.0344 |
| 23 | 0.0301 | 16 | 0.0485 | 20 | 0.0134 |
| 24 | 0.0109 | 17 | 0.0326 | 23 | 0.0108 |
| 26 | 0.0150 | 19 | 0.0836 | 25 | 0.0148 |
| 49 | 0.0038 | 23 | 0.0147 | 37 | 0.0040 |
| 51 | 0.0054 | 116 | 0.0008 | 41 | 0.0059 |
| 61 | 0.0023 | 145 | 0.0013 | 49 | 0.0016 |
| 69 | 0.0111 | 164 | 0.0009 | 63 | 0.0011 |
| 74 | 0.0029 | 201 | 0.0009 | 69 | 0.0040 |
| 78 | 0.0048 | 270 | 0.0051 | 78 | 0.0008 |
| 84 | 0.0033 | 298 | 0.0021 | 94 | 0.0020 |
| 90 | 0.0052 | 364 | 0.0099 | 98 | 0.0013 |
| 93 | 0.0034 | 375 | 0.0019 | 101 | 0.0025 |
| 123 | 0.0083 | 394 | 0.0073 | 104 | 0.0014 |
| 160 | 0.0041 | 418 | 0.0027 | 111 | 0.0024 |
| 207 | 0.0055 | 500 | 0.0016 | 114 | 0.0014 |
| 224 | 0.0139 | 1.62 g_rms_ | | 117 | 0.0020 |
| 245 | 0.0031 |  | | 121 | 0.0012 |
| 276 | 0.0129 |  |  | 139 | 0.0024 |
| 287 | 0.0036 |  |  | 155 | 0.0021 |
| 353 | 0.0027 |  |  | 161 | 0.0034 |
| 375 | 0.0049 |  |  | 205 | 0.0042 |
| 500 | 0.0010 |  |  | 247 | 0.0303 |
| 2.20 g_rms_ | |  |  | 257 | 0.0027 |
|  | |  |  | 293 | 0.0092 |
|  |  |  |  | 330 | 0.0116 |
|  |  |  |  | 353 | 0.0231 |
|  |  |  |  | 379 | 0.0083 |
|  |  |  |  | 427 | 0.0220 |
|  |  |  |  | 500 | 0.0014 |
|  |  |  |  | 2.05 g_rms_ | |

**Table S2**. Vibration test conditions for combined wheeled vehicle transportation. Test directions：Vertical direction, Transverse directions, Longitudinal direction;Test time：20min/ direction

| Wave Form | Acceleration peak | Pulse width | Numbers of impact | Test directions |
| --- | --- | --- | --- | --- |
| Final peak saw tooth wave | 20g | 11ms | 3 times/direction | Three axis |

**Table S3**. The conditions of Impact test.


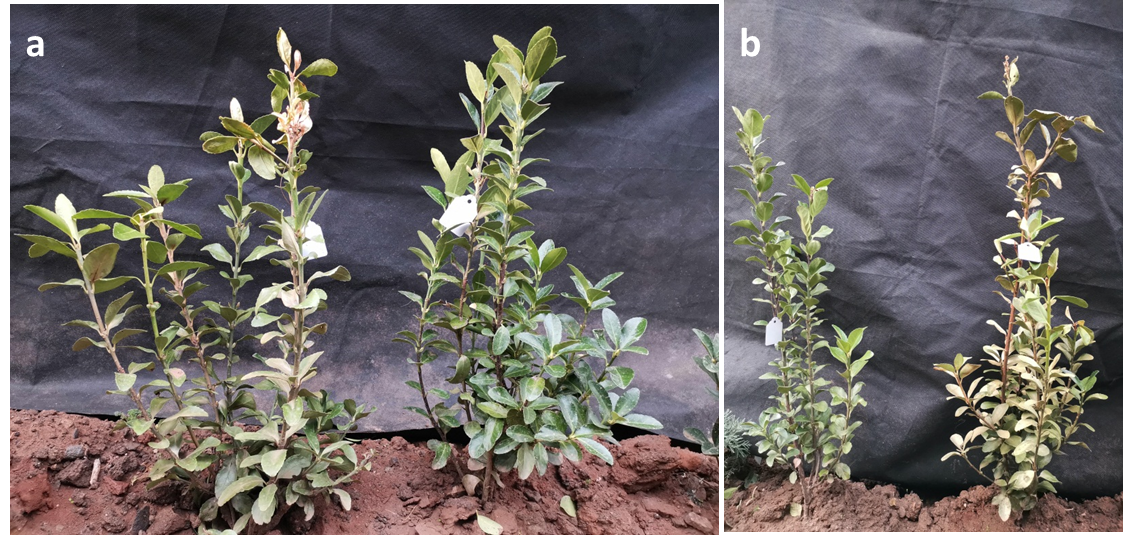


**Figure S1** Changes of transplanted seedlings when the ternary blend adhesive contacted the roots of transplanted seedlings: the left seedling in the (a) and the right seedling in the (b)


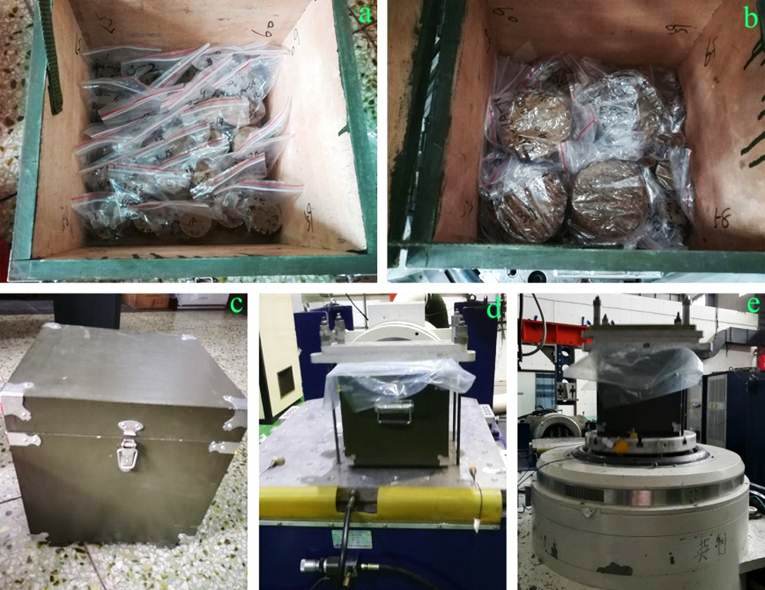


**Figure S2** Vibration tests of 35 soil columns on expressway transportation
